# Catecholaminergic Polymorphic Ventricular Tachycardia in a 16-year-old: Case Report

**DOI:** 10.5811/cpcem.34847

**Published:** 2025-04-26

**Authors:** John Wahhab, Ani Oganesyan, Krishi Korrapati

**Affiliations:** Chicago Medical School at Rosalind Franklin University of Medicine and Science, North Chicago, Illinois

**Keywords:** case report, catecholaminergic polymorphic ventricular tachycardia, arrhythmia

## Abstract

**Introduction:**

Catecholaminergic polymorphic ventricular tachycardia (CPVT) is a rare, inheritable cardiac disorder associated with stress- or exercise-induced syncope or cardiac arrest in children and young adults. Diagnosis of CPVT is often missed or delayed due to variable presentation and normal cardiac imaging and electrocardiogram results, with about 40% of patients dying within 10 years of diagnosis. This case underscores the importance of cross-departmental communication when managing complex pediatric cases, especially when using an interpreter.

**Case Report:**

A 16-year-old male presented to the hospital with cardiac arrest in ventricular fibrillation following collapse despite a history of treatment with flecainide and nadolol. He was resuscitated, stabilized with antiarrhythmic drips, received an implantable cardioverter defibrillator, and was discharged neurologically intact nine days later. It is vital for physicians to consider CPVT in young patients with syncope to prevent errors in diagnosis of this highly fatal disease.

**Conclusion:**

Catecholaminergic polymorphic ventricular tachycardia is a rare genetic disease with significant morbidity and mortality. Treatment decisions for acute CPVT often occur without prior knowledge of the disease; so, in patients diagnosed with CPVT, physicians should implement appropriate therapeutic options to prevent future cardiac events. For patients who remain symptomatic despite compliance with beta blockers and/or other antiarrhythmic therapy, interventions such as placement of an implantable cardioverter defibrillator or sympathetic denervation may be necessary to prevent life-threatening arrhythmias. This case also underscores the importance of obtaining a detailed family history and coordinating care with other physicians in cases where history is limited.

## INTRODUCTION

Here we present the case of a 16-year-old male who experienced sudden cardiac arrest because of catecholaminergic polymorphic ventricular tachycardia (CPVT), an uncommon congenital and life-threatening condition. The patient was successfully resuscitated and stabilized with Advanced Cardiovascular Life Support (ACLS) and multiple cardiac medications, including a transition from amiodarone to esmolol following consultation with his cardiologist. Although CPVT is uncommon, it is crucial for emergency physicians to recognize and manage it promptly. Our case underscores the importance of early identification and appropriate intervention, which led to the patient’s complete neurological recovery and successful placement of an implantable cardioverter defibrillator (ICD).

## CASE REPORT

A 16-year-old boy presented to the emergency department (ED) via emergency medical services (EMS) in cardiac arrest, receiving active chest compressions. Per EMS, he had collapsed suddenly and was found pulseless with no spontaneous respiration and cardiopulmonary resuscitation was immediately started. En route, the patient was in ventricular fibrillation (VF), defibrillated five times, and given 300 milligrams of amiodarone intravenously (IV) and epinephrine IV per ACLS protocol. He was intubated in the ED, where ACLS was continued and return of spontaneous circulation was achieved. Vitals showed a temperature 37 °Celsius, heart rate 110 beats per minute, respirations 18 breaths per minute, 100% oxygenation on the ventilator, and blood pressure 71/51 millimeters of mercury. His exam showed no trauma, a temporary airway in the oropharynx, and mechanical breath sounds bilaterally.

The patient was intubated with a 7.5 endotracheal tube and started on an amiodarone drip due to initial ventricular tachycardia. Due to hypotension, he was started on a norepinephrine drip, along with fentanyl and propofol for pain and sedation. The patient’s electrocardiogram (ECG) showed wide complex tachycardia but no ST-segment elevation myocardial infarction. His mother, using an interpreter, reported he had been in a normal state of health before collapsing and had a history of “bradycardia,” taking flecainide and nadolol for it. He had not been sick and was not exercising when it happened. Although an interpreter was used, history was limited due to a language barrier and limited medical literacy of the family.

His labs showed significantly elevated troponin at 344 picograms per milliliter (pg/mL) (reference range 0–40 pg/mL); aspartate aminotransferase of 341 units per liter (U/L) (8–33 U/L) and alanine transaminase of 404 U/L (7–56 U/L); potassium of 3.5 milliequivalents per liter (mEq/L) (3.4–4.7 mEq/L); magnesium of 2.6 mEq/L (1.3–2.1 mEq/L); bicarbonate of 17 mEq/L (22–29 mEq/L), an anion gap of 23 millimoles per liter (mmol/L) (4–12 mmol/L); and negative acetaminophen, salicylates, alcohol and urine drug screen. Due to the history of flecainide use, a possible overdose was considered, and poison control recommended a bicarbonate drip due to the wide complex tachycardia. After consulting the patient’s cardiologist, who revealed a history of CPVT, the amiodarone drip was changed to esmolol. They recommended strict electrolyte repletion with goal potassium greater than four mEq/L and magnesium of greater than two mEq/L. The patient was given 40 mEq of potassium chloride intravenous.

As recommended by the cardiologist, the patient was transferred to their facility via critical care ambulance. While in the pediatric intensive care unit, he was started on milrinone, lidocaine, and esmolol drips to stabilize his heart rhythm. On hospital day two he was extubated, had an ICD placed, and was weaned off the drips, starting oral nadolol and flecainide. He developed a post-ICD deep vein thrombosis, which was treated with anticoagulation. He was discharged on hospital day 10 with a completely normal neurological exam. His post-ICD stress test was normal with no arrhythmia, and he was eventually taken off anticoagulation. On subsequent follow-up visits the patient was noted to be doing well on his current medications.

**CPC-EM Capsule**

What do we already know about this clinical entity?

*Catecholaminergic polymorphic ventricular tachycardia (CPVT) is an inherited heart condition that causes life-threatening arrhythmias, especially during periods of stress, due to a genetic mutation in calcium regulation*.

What makes this presentation of disease reportable?

*When CPVT causes cardiac arrest, it is usually undiagnosed. Here, it was already known, but a language barrier prevented the family from disclosing this information*.

What is the major learning point?

*Detailed family history, a broad differential, and interpreter use is integral in identifying rarer cardiac diagnoses to provide optimal care*.

How might this improve emergency medicine practice?

*Direct physician-to-physician communication and a broad differential may reduce risk of diagnostic error, improving treatment in acute cardiac settings*.

## DISCUSSION

Catecholaminergic polymorphic ventricular tachycardia is a rare heart condition characterized by polymorphic or bidirectional tachycardia that is mostly inherited in an autosomal dominant manner. It is often undiagnosed due to normal ECG and postmortem findings. The disease has a heterogeneous genetic basis, typically involving ryanodine or calsequestrin channel mutations. These mutations lead to increased calcium leakage from the sarcoplasmic reticulum during diastole, causing an abnormal elevation in intracellular calcium levels, which can result in VF. Episodes are often precipitated by acute exercise or emotion and can lead to syncope and potentially fatal VF. The age of onset is most frequently between 7–12 years, although there have been cases of onset in the forties. Diagnosis is defined by a structurally normal heart, normal ECG, or exercise- or stress- induced VT or those with certain genetic variants. The differential diagnosis for arrhythmogenic syncope in a pediatric patient includes Brugada syndrome, congenital prolonged QT-interval, hypertrophic obstructive cardiomyopathy, Wolff-Parkinson-White syndrome, and arrhythmogenic right ventricular cardiomyopathy.

The true prevalence of CPVT is likely higher because these other inherited arrhythmias can at least present with resting ECG abnormalities. Treatment includes resuscitation, beta blockers, flecainide, nadolol and, in refractory cases, calcium channel blockers, ICD implantation, and left cardiac sympathetic denervation.^2^ This case highlights the clinical, diagnostic, and management challenges of CPVT in pediatric patients.^3^ The patient presented with cardiac arrest due to VF despite treatment with flecainide and nadolol. Physicians must recognize the nuances of this case to be able to recognize and diagnose CPVT in patients with unexplained syncope or cardiac events, even with ongoing antiarrhythmic therapy.

Catecholaminergic polymorphic ventricular tachycardia has a variable clinical presentation and no unique ECG findings. Electrocardiograms are typically normal until an arrhythmic episode occurs, showing polymorphic VT, bidirectional tachycardia, or supraventricular tachycardias.^4^,^5^ Yet these findings can also apply to other cardiac disorders such as nonischemic cardiomyopathies.^6^ Our patient had wide complex tachycardia, which suggests many other diagnoses, including Brugada syndrome and drug overdose.^6^ In turn, accurate diagnosis and management depend on clinical suspicion and thorough evaluation of the patient›s history and presentation.

Our initial suspicion in this case was a potential overdose on a medication like flecainide that can induce VT, but this was a red herring. The discovery that the patient had CPVT highlights the importance of detailed family history despite communication barriers and knowledge gaps, as well as interdisciplinary collaboration to identify CPVT before it presents as cardiac arrest. We recommend direct physician-to-physician communication when possible to clarify any gaps in knowledge in patients with complex medical histories, as well as always using a licensed interpreter.

## CONCLUSION

In the absence of treatment, CPVT is a highly lethal arrhythmic disorder. To maintain hemodynamic stability and minimize the risk of sudden cardiac death, physicians treating patients presenting to the ED with cardiac arrest must recognize this condition quickly. Nevertheless, patients may still experience symptoms even when diagnosed and compliant with maximally tolerated medical therapy. In describing this case, we aimed to contribute to the literature describing these cases and enhance awareness of this condition. It is critical that physicians be aware of the possibility of CPVT in cardiac arrest with apparent arrhythmic control, ask for a detailed history, and collaborate with other emergency clinicians when unable to obtain clear information from the family before arrest. A further aim was to emphasize the consideration of concurrent interventions, such as ICDs or sympathetic denervation, in high-risk patients to reduce the risk of adverse cardiac events.
